# A Highly Specific Holin-Mediated Mechanism Facilitates the Secretion of Lethal Toxin TcsL in *Paeniclostridium* *sordellii*

**DOI:** 10.3390/toxins14020124

**Published:** 2022-02-08

**Authors:** Callum J. Vidor, Audrey Hamiot, Jessica Wisniewski, Rommel A. Mathias, Bruno Dupuy, Milena Awad, Dena Lyras

**Affiliations:** 1 Infection and Immunity Program, Monash Biomedicine Discovery Institute and Department of Microbiology, Monash University, Clayton, VIC 3800, Australia; callum.vidor@monash.edu (C.J.V.); jess.wisniewski@monash.edu (J.W.); rommel.mathias@csl.com.au (R.A.M.); milena.awad@monash.edu (M.A.); 2 School of Biological Sciences, Monash University, Clayton, VIC 3800, Australia; 3 Laboratoire Pathogenèse des Bactéries Anaérobies, UMR-CNRS 6047, Institut Pasteur, Université de Paris, F-75015 Paris, France; Audrey.Hamiot@inrae.fr (A.H.); bruno.dupuy@pasteur.fr (B.D.); 4 Infection and Immunity Program, Monash Biomedicine Discovery Institute and Department of Biochemistry and Molecular Biology, Monash University, Clayton, VIC 3800, Australia

**Keywords:** *Paeniclostridium sordellii*, *Clostridioides difficile*, large clostridial glucosylating toxins, toxin secretion, holins, protein secretion

## Abstract

Protein secretion is generally mediated by a series of distinct pathways in bacteria. Recently, evidence of a novel bacterial secretion pathway involving a bacteriophage-related protein has emerged. TcdE, a holin-like protein encoded by toxigenic isolates of *Clostridioides difficile*, mediates the release of the large clostridial glucosylating toxins (LCGTs), TcdA and TcdB, and TpeL from *C. perfringens* uses another holin-like protein, TpeE, for its secretion; however, it is not yet known if TcdE or TpeE secretion is specific to these proteins. It is also unknown if other members of the LCGT-producing clostridia, including *Paeniclostridium sordellii* (previously *Clostridium sordellii*)*,* use a similar toxin-release mechanism. Here, we confirm that each of the LCGT-producing clostridia encode functional holin-like proteins in close proximity to the toxin genes. To characterise the respective roles of these holin-like proteins in the release of the LCGTs, *P. sordellii* and its lethal toxin, TcsL, were used as a model. Construction and analysis of mutants of the *P. sordellii tcsE* (holin-like) gene demonstrated that TcsE plays a significant role in TcsL release. Proteomic analysis of the secretome from the *tcsE* mutant confirmed that TcsE is required for efficient TcsL secretion. Unexpectedly, comparative sample analysis showed that TcsL was the only protein significantly altered in its release, suggesting that this holin-like protein has specifically evolved to function in the release of this important virulence factor. This specificity has, to our knowledge, not been previously shown and suggests that this protein may function as part of a specific mechanism for the release of all LCGTs.

## 1. Introduction

Protein secretion, defined as the transport of proteins from the cell cytoplasm to the extracellular milieu or into a target cell [1], is a critically important process in all bacteria. In pathogenic bacteria, the substrates of protein secretion systems are often virulence factors that induce host damage or cellular changes [2]. For diderm (Gram-negative) bacterial cells, the secretion process involves proteins crossing the cytoplasmic membrane (CM), transferring through the periplasm, and then traversing the outer-membrane [1]. To achieve this complex process, diderm bacteria have developed a number of distinct secretion systems, with nine major types currently defined [3]. In monoderm (Gram-positive) bacterial cells, this process appears to be simpler since proteins must only cross a single cellular membrane to be secreted [1]. Once past the CM, secreted proteins often passively diffuse through the peptidoglycan layer [2]. Most proteins secreted by monoderms utilise two main pathways to cross the CM: the general secretion (Sec) or the twin arginine translocation (Tat) pathway [4]. Although there are features common to both pathways, the transport mechanism of each is different, with the Sec pathway transporting unfolded proteins, whereas the Tat pathway translocates folded proteins [2,4]. Transport by either system requires that the substrate protein contains a specific secretion signal recognition sequence, or signal peptide, at its N-terminus [2]. 

Many pathogenic bacteria utilise protein secretion mechanisms to release potent exotoxins that damage the host [2]. The clostridia, which are Gram-positive, strictly anaerobic, spore forming bacteria, are one such genus [5]. Some clostridial exotoxins appear to have signal peptides to facilitate their release from the cell [6,7,8]; however, others do not have any identifiable signal peptides, and the secretion process for these is not known [9,10,11]. Large clostridial glucosylating toxins (LCGTs) belong to the latter group. LCGTs are large (191–308 kDa) single-chain toxins [11,12] that function in mammalian target cells by glucosylating small GTPases, usually of the Rho or Ras family, leading to cytoskeletal rearrangement, cell rounding, and death [13]. LCGTs include TcdA and TcdB from *Clostridioides difficile*, TcsH (haemorrhagic toxin) and TcsL (lethal toxin) from *Clostridium sordellii* (renamed *Paeniclostridium sordellii*), TpeL from *Clostridium perfringens*, and Tcnα (alpha toxin) from *Clostridium novyi* [11,12]. The spectrum of diseases associated with these toxins is broad and depends on the site of infection and on differences in the specific cellular receptors and GTPase targets for each toxin [13,14]. 

**Figure 1:**
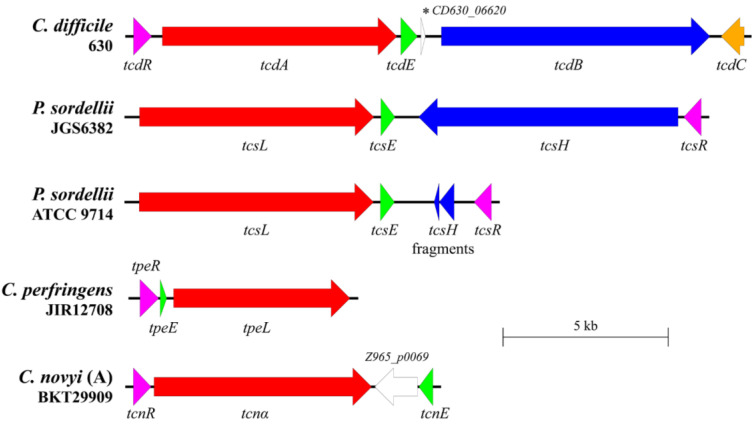
The PaLoc-like regions encoding clostridial LCGTs. Shown are the ORFs encoded within the PaLocs of *C. difficile*, *P. sordellii*, *C. perfringens* and *C. novyi*. Strain names are indicated under each species. Genes encoding orthologous products are indicated with the same colour: *tcdB*-like LCGT, red; *tcdA*-like LCGT, dark blue; alternative sigma factor/positive regulator, purple; transcriptional repressor, orange; holin-like protein, green; other ORFs, white. Produced using EasyFig [26].

In *C. difficile*, the LCGTs are encoded at a defined chromosomal site called the Pathogenicity Locus (PaLoc) (Figure 1) [15]. As well as the genes *tcdA* and *tcdB*, PaLoc encodes the accessory genes *tcdR, tcdC* and *tcdE* [15]. TcdR is an alternative sigma factor and a positive regulator of PaLoc genes [16,17], whereas TcdC appears to be an anti-sigma factor that negatively regulates toxin production [18,19], although this is contentious [20]. As for all LCGTs, TcdA and TcdB do not carry any recognisable signal peptides to indicate that they are secreted by one of the classical pathways. However, the holin-like protein TcdE seems to play a major role in their secretion [10], although a coexisting bacteriolytic mechanism also facilitates the release of the toxins in vitro, under certain growth conditions [21]. Furthermore, it has recently been shown that TpeE, a holin-like protein, is required for the secretion of the *C. perfringens* LCGT TpeL, which reinforces the role of a holin-dependent secretion system [22]. Holins are transmembrane proteins associated with the lytic lifecycle of bacteriophages. At the time of viral exit, holins will oligomerise in the CM, forming a pore through which a peptidoglycan-degrading endolysin transits, thereby compromising the bacterial cell wall and resulting in cellular lysis and bacteriophage release [23]. Despite holins being associated with cell lysis mechanisms [23,24], the holin-mediated release of *C. difficile* LCGTs is non-lytic [10,25].

More broadly, the role of holins in protein secretion is controversial [27]. Phage-encoded holins have been implicated in the increased release of extracellular toxins, such as SheA and Stx1 in *Escherichia coli* [28,29]. Furthermore, bacterially encoded holin-like proteins have been shown to mediate chitinase release in *Serratia marcescens* [30]. One reason why holin-mediated systems may not be fully accepted as true bacterial secretion systems is that, with respect to phages, the roles of holins is to passively enable the transfer of folded endolysins into the periplasm [27], resulting in cellular lysis [23]. Neither of these processes are associated with specificity of protein release. Despite this, in the bacterial-associated holin-secretion mechanism, studies have observed the non-lytic nature and lack of cytoplasmic leakage [10,30], while the specificity of such a system has not yet been conclusively validated. This study aimed to extend investigation of the secretion mechanisms associated with the LCGTs encoded by a number of pathogenic clostridial species and their specificity using *P. sordellii* as a model organism. Here, we show that the *P. sordellii* LCGT TcsL requires a holin, TcsE, for efficient secretion, and that TcsL appears to be the only protein secreted by TcsE in *P. sordellii*, suggesting that the holin-mediated release for the clostridial LCGTs is highly specific. 

## 2. Results

### 2.1. Putative Holins Are Encoded within the PaLoc Regions of Pathogenic Clostridial Species

**Figure 2:**
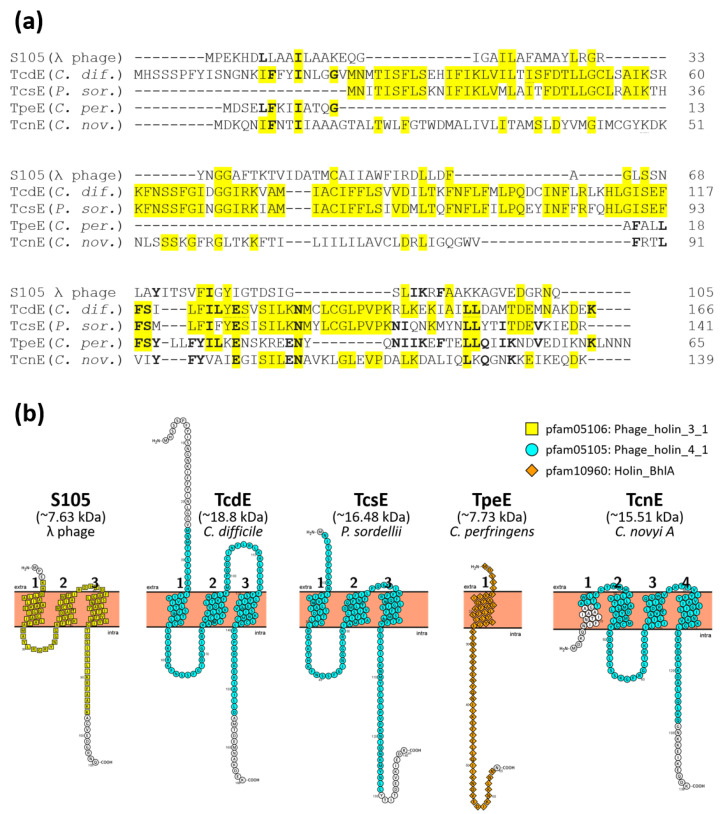
Bioinformatic analysis of predicted holin proteins encoded by clostridial PaLoc regions. (**a**) Alignment of clostridial holin-like proteins from *C. difficile* (AJP10337), *P. sordellii* (CEJ75464), *C. perfringens* (CRG98304) and *C. novyi* type A (KEH84763) with λ phage holin S105 (UniProtKB-P03705). Amino acids with identity to *C. difficile* TcdE are highlighted in yellow; those identical to *C. perfringens* TpeE are bolded. Constructed using Clustal Omega from the EMBL-EBI suite [33]. (**b**) Predicted membrane topology and conserved domains of clostridial holins. The predicted molecular weight in kDA is displayed for each holin. Cell membranes are represented as solid pink bars in which transmembrane domains (TMDs) are embedded. The number of TMD domains are shown in black. No signal peptides were predicted for any of the sequences. Amino acids that correspond to conserved protein domains are indicated as follows: yellow squares—pfam05106 domain, Phage_holin_3_1; blue circles—pfam05105 domain, Phage_holin_4_1; orange diamonds—pfam10960 domain, Holin_BhlA (found in a number of holin-like proteins of phage and bacterial origin. BhlA displays auto-lytic activity leading to bacterial cell death by membrane disruption) [34,35]. Figure constructed using Protter [36].

All LCGT-encoding PaLoc regions of the pathogenic clostridia [15,22,31,32] encode homologues of TcdE and the alternative sigma factor TcdR (Figure 1). The *C. novyi* PaLoc also encodes homologues of *tcdE* (*tncE*) and *tcdR* (*tcnR*), shown here by examining the closed phage genome from strain BKT29909 (Figure 1). These holin-like proteins vary between the clostridia, both in their predicted molecular weights and transmembrane domains (Figure 2). Furthermore, apart from TcdE and TcsE which appear related, there is limited protein identify among the clostridial holin proteins (Figure 2). However, the presence of holin-like proteins encoded by each of these clostridial PaLocs suggests that they may play a similar role to that of TcdE and therefore act to release their respective LCGTs. This suggestion is again supported by the recent finding that TpeE from *C. perfringens* controls the secretion of the LCGT TpeL, despite the obvious difference in structure and identify to the other clostridial holins (Figure 2) [22]. It has been noted that TpeE has more similarity to TatA-like holins, displaying 89% identify to the holin UviB from *C. perfringens* [22].

### 2.2. TcsE, TpeE and TcnE Function Like TcdE as Holins

**Figure 3:**
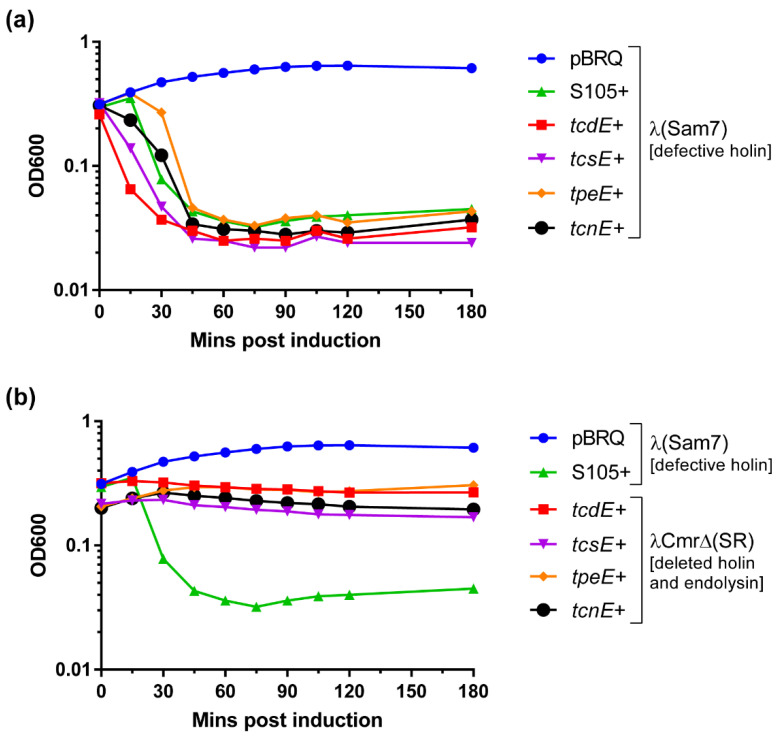
Determination of holin function of TcdE-like proteins in *E. coli*. Lysis curves after thermoinduction of lysogenic cultures of *E. coli* λcI_857_Sam7 (**a**) or *E. coli* λCmrΔ(*SR*) (**b**) carrying plasmids expressing TcdE, TcsE, TpeE, or TcnE *in trans*. A lysogenic strain carrying pJN5 encoding the λ holin S^105^, and pBRQ(ΔRBS) were used as positive and negative controls, respectively. The cultures were grown at 30 °C until OD_600_ = 0.15–0.20, thermoinduced for 5 min at 42 °C, and then incubated at 37 °C, with OD_600_ measurements taken every 15 min.

We evaluated the holin-like activity of TcsE, TpeE, and TcnE by complementing an *E. coli* λ lysogen defective for the S^105^ holin with their respective encoding genes, as previously performed for TcdE and UviB [10,37]. The *tcsE*, *tpeE*, and *tcnE* genes were cloned into the heat-inducible expression plasmid pBRQ(ΔRBS) and were introduced into *E. coli* MC1061 carrying the λ lysogen (cI_857_Sam7), which has a functional endolysin gene but a nonsense mutation in its holin gene. When the λ holin S^105^ gene is used to complement the holin gene mutation in this strain, bacterial lysis is completed 45 min after heat induction (Figure 3a) since the presence of a functional holin allows the endolysin to cross the membrane and degrade murein from the outside, resulting in cell lysis. No lysis was observed in the presence of the vector alone (Figure 3a). When TcsE, TpeE, and TcnE were expressed in *E. coli* strain MC1061(λcI_857_Sam7), complete bacterial lysis was seen ~45 min after heat induction, similar to the positive control (λ holin S^105^) and to TcdE (Figure 3a). Expression of TcsE, TpeE, and TcnE in the *E. coli* strain λCmrΔ(*SR*), which has deletions in both the holin and endolysin genes, did not cause cell lysis (Figure 3b), confirming that lysis in the strain MC1061(λcI_857_Sam7) resulted specifically from holin-facilitated endolysin activity, demonstrating that these proteins function in the same way as a phage holin, similar to TcdE [10]. This finding also supports the role of TpeE as a holin protein [22], which, until now, had not been shown to complement holin functionality. 

### 2.3. The Release of TcsL in P. sordellii Is Facilitated by the PaLoc-Encoded Holin TcsE

To determine the roles of the PaLoc-associated holin gene in LCGT secretion, we focused on the role of TcsE in the secretion of TcsL. Two independent *P. sordellii* strain ATCC 9714 *tcsE* mutants were constructed using the TargeTron insertional inactivation system. The mutations were confirmed using PCR (data not shown) and Southern hybridisation (Figure S1) and were complemented *in trans* through the introduction of wild-type *tcsE* cloned in the clostridial vector pRPF185, under the control of a Tet inducible promoter. The wildtype, mutant, and complemented isogenic panel of strains were then tested for their ability to release TcsL. To do this, *P. sordellii* strains at the mid-exponential growth phase were diluted to a low optical density in TY broth (a media deficient in glucose, as the sugar is known to repress TcsL expression [32]) and grown for either 10 or 22 h. At one hour and five hours into their growth, respectively, anhydrous tetracycline was added to all cultures to induce expression of the cloned genes in the complementation strains. At ten hours, cells were collected for extraction of RNA, followed by collection and filter sterilisation of the culture supernatant at 10 h and 22 h post inoculation. 

**Figure 4:**
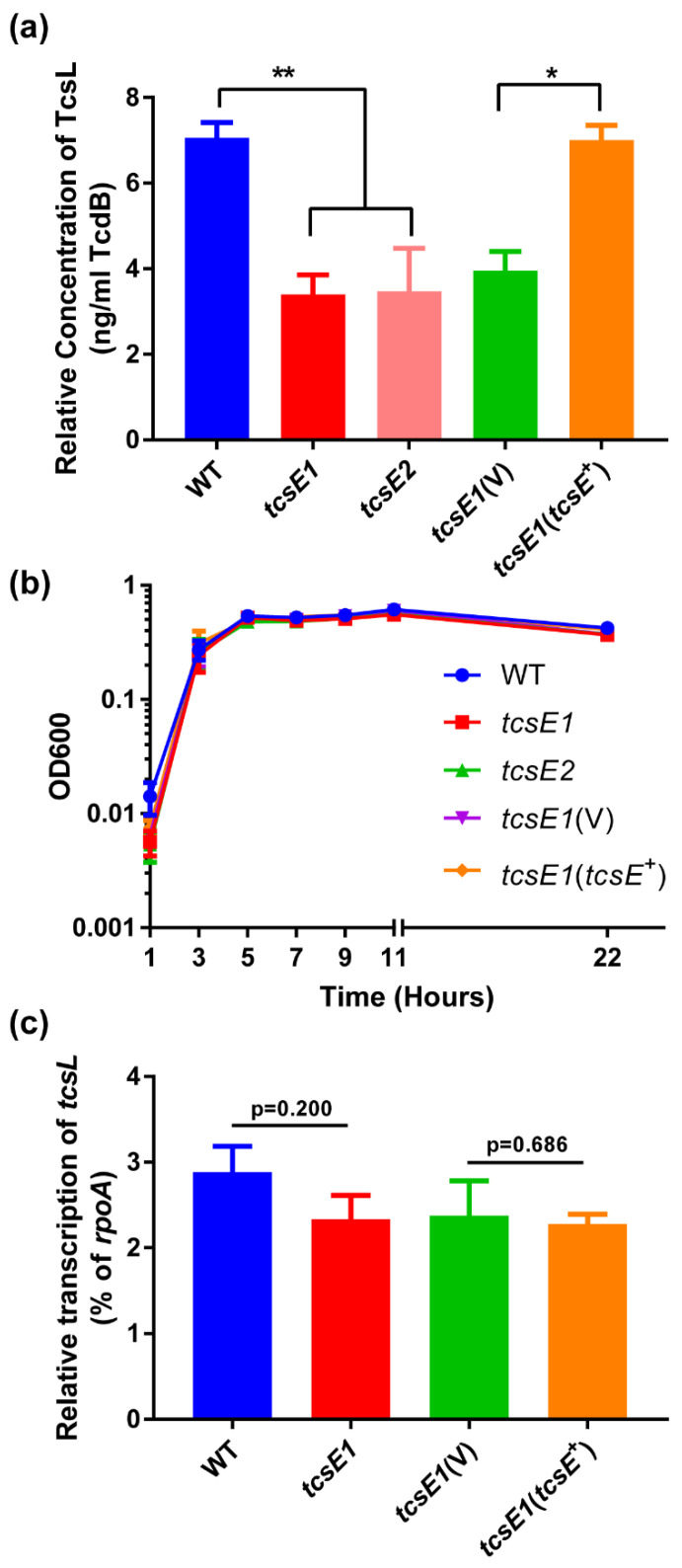
Analysis of TcsL secretion in a holin mutant of *P. sordellii*. (**a**) Levels of TcsL in the TY culture supernatant of the isogenic strains. At T = 22 h, culture supernatants were collected and assayed using a TcdB-specific ELISA detection kit (tgcBiomics). The concentrations of TcsL detected relative to a TcdB standard protein curve are shown, having been standardised to total protein concentration in the supernatant as determined by a BCA assay. n ≥ 4, error bars indicate SEM. Statistical analysis was conducted using a Mann–Whitney *U* Test * *p* < 0.05, ** *p* < 0.01. (**b**) Growth curve of the TY cultures over 22 h. After dilution in TY broth, an OD_600_ reading was taken at T = 1, followed by every 2 h until T = 11. A final reading was taken at T = 22. n ≥ 3, error bars indicate SEM. (**c**) Relative levels of *tcsL* transcription among the isogenic panel of mutants. At T = 10 h, cells were collected from TY cultures; RNA was extracted and cDNA synthesized. Concentrations of *tcsL* and *rpoA* (housekeeping control) transcript were determined from the cDNA using ddPCR. Displayed is the relative expression of *tcsL* as a percentage of *rpoA* (housekeeping gene) expression for each mutant. n = 4, error bars indicate SEM. Statistical analysis was conducted using a Mann–Whitney *U* Test.

TcsL levels were determined using a *C. difficile* TcdB-specific ELISA since the TcdB antibodies in this ELISA cross-react with TcsL (Figure S2). Both independent mutants of *tcsE* had ~50% less TcsL in their supernatant fractions compared to the wild-type strain (Figure 4a), which was restored upon *in trans* complementation with wild-type *tcsE*, but not with the vector alone (Figure 4a). These results showed a similar trend when the supernatants were tested at 10 h; however, toxin levels at this time point were low (Figure S3). To validate that functional toxin was being released, a Vero cell cytotoxicity assay was performed using concentrated culture supernatants. This experiment confirmed that the toxin released was indeed functional and supports the results obtained with the ELISAs (Figure S4). Furthermore, the reduction in TcsL levels between strains did not result from any alterations in growth patterns (Figure 4b) or differences in *tcsL* expression (Figure 4c); thus, the holin-like protein TcsE appears to be required for the efficient release of TcsL from *P. sordellii*. Since TcsL is still detected in the supernatants of the *tcsE* mutants (Figure 4a), concomitantly to the cell lysis that is observed during the stationary growth phase (Figure 4b), this result suggests a co-existing lytic mechanism for LCGT release, as is observed for *C. difficile* [21].

### 2.4. The TcsE Holin-like Protein Is Specific for TcsL Release Alone 

TcsE clearly plays a major role in the secretion of TcsL in *P. sordellii*, but it is not known if TcsE facilitates the release of any other proteins. To investigate this hypothesis, secretome samples of each of the *tcsE* isogenic panel of strains were collected (in four biological replicates per strain). This was achieved through harvesting the culture supernatants of the 22-h timepoint of each strain from the analysis represented in Figure 4, followed by enzymatic digest and collection of all peptides. The 22 h timepoint was chosen as the timepoint for analysis since TcsL levels are very low earlier in the growth phase, making differentiation of its secretion from other supernatant proteins difficult. 

**Figure 5:**
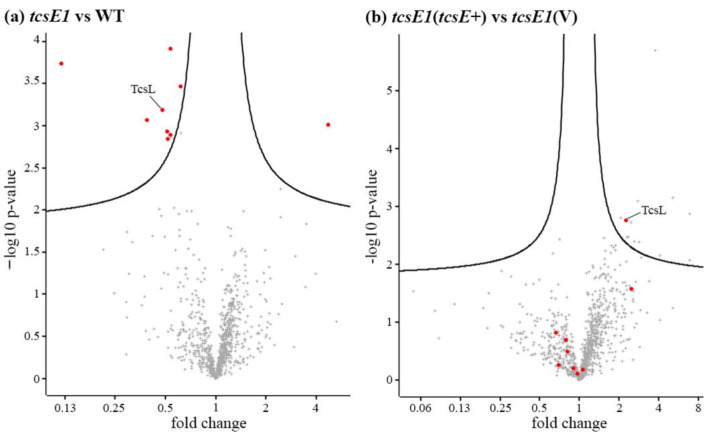
Secretome analysis of the *tcsE* holin mutant of *P. sordellii*. Shown are volcano plots representing a label-free quantification nLC-MS/MS analysis, comparing the abundance of individual proteins in the culture supernatant of wild type (WT) *P. sordellii* ATCC9714 with an isogenic *tcsE* mutant (**a**), as well as comparing the complemented *tcsE* mutant against the same mutant carrying the control vector pRPF185 (**b**). Each data point represents an individual protein detected in the supernatant. The x-axis displays the fold change between the means of the two strains. The y-axis displays the −log10 of the *p*-value from a 2-tailed student’s *t*-test on log2 transformed values. The threshold for significance was determined as a permutation-based false discovery ratio of 0.1 combined with an artificial within group’s variance of 0.1 using 250 randomisations, which is represented as the black curves on the chart. The nine proteins that have significantly different levels in the supernatant between the WT and the mutant are shown as red circles on both plots.

Proteins were identified using mass spectrometry, and their levels determined by label-free quantification. Proteins were included in the analysis if two unique peptides were detected in at least three biological replicates of one of the four test strains. In total, 9 out of the total 802 proteins were identified to have significantly altered levels in the culture supernatant from the *tcsE* mutant based on a false discovery ratio of 0.1 with 250 permutations combined with a within-group variance cutoff of 0.1 (Table 1, represented as red circles in Figure 5a). This cutoff was chosen due to a likelihood of lysed cell cytoplasm components partially clouding secretion profiles, is represented as black curves in Figure 5, and equates approximately a *p*-value cutoff of >0.01 combined with an approximate fold increase greater than 2 (positive or negative) between means. Proteins with reduced abundance were represented by the toxin TcsL, a probable polysaccharide-deacetylase, a phospholipase D-nuclease domain protein, and a group of protein constituents from the 50S ribosome (Table 1a). However, the vast majority of predicted cytoplasmic proteins identified via mass spectrometry (File S1, Figure 5a) were not significantly altered between the WT and *tcsE* mutant strains; therefore, we suggest that differences between these strains are unlikely to be due a general lytic mechanism of TcsL release. 

To confirm the role of *tcsE* in the differential release of these nine proteins, the secretome of the complemented *tcsE* mutant was compared to the vector control of the *tcsE* mutant (Figure 5b). In this comparison, 13 proteins were determined to be significantly altered in abundance (Figure 5b, Table 1b), using the same statistical/foldchange cutoff. However, TcsL was the only protein to be significantly altered in both comparisons (Table 1, Figure 5), displaying a ~50% reduction in the *tcsE* mutant in comparison to the wild-type strain, which was restored upon complementation (Table 1, Figure 5). We suggest that the remaining 8 proteins identified from the M vs. WT and 12 proteins from the C vs. VC (Table 1) comparisons were aberrations introduced by unrelated factors such as minor differences in growth and lytic release of proteins unrelated to TcsE. Of these 20 proteins, only three display *p*-values that could be considered significantly different between comparisons (both *p* < 0.05), those being the phospholipase D nuclease domain protein from the first comparison (Table 1a), the putative delta-lactam-biosynthetic de-N-acteylase, and a conserved hypothetical protein from the second (Table 1b). If the difference in the proteins levels between treatments was truly due to the presence/absence of TcsE, we would expect a reciprocal pattern when comparing the mutant to the wildtype of that when comparing the vector control to the complemented strain (e.g., a decrease vs. an increase). However, the differences in these three proteins are unlikely to be due to the action of TcsE, as they are not reciprocal in their abundance between the WT vs. MT and the C vs. VC comparison. They are either increased in both the mutant and the complemented strain, or they are both reduced in abundance, suggesting that the presence of TcsE is not controlling any differences in abundance. As TcsL is the only protein that appeared in the cutoff in both comparisons (Table 1, Figure 5) and displays a reciprocal pattern between the M vs. WT and V vs. VC comparisons (0.48 vs. 2.25, Table 1), we suggest that the TcsE-mediated secretion of TcsL is specific, being the only protein released by this holin-like protein under both conditions tested.

## 3. Discussion

The best-understood bacterial secretion systems in monoderm organisms, such as Tat and Sec, are highly specific, with amino acid signals within the secreted proteins being recognised by an export machinery for transport out of the bacterial cell [2]. In addition to these signal peptide-driven secretion machineries, recent studies have suggested that unrelated secretion systems function to export a diverse range of products made within bacterial cells. One of these systems consists of one or two components, a holin and/or an endolysin [10,30,38,39]. These systems, however, are not well accepted as specific secretion systems, partly because substrate specificity has not been clearly demonstrated, and because they are thought to facilitate protein secretion through a generalised cell lysis mechanism [27]. The work presented here definitively shows that at least a component of one such system, mediated by the *P. sordellii* holin TcsE, functions to export TcsL and that the release of this toxin occurs independently of other bacterial proteins, suggesting that TcsL is the only protein dependent on this export system for its release in its natural host.

In *C. difficile*, using almost identical strains, the holin-like protein TcdE was shown to mediate the release of the LCGTs TcdA and TcdB in one study [10], but another study suggested that TcdE was not involved in this phenotype [40]. Recent work has clarified the difference between the two earlier studies by proposing that two distinct processes are involved in LCGT release in *C. difficile* strain 630 according to the in vitro medium used. The first of these is a lytic mechanism controlled by the lytic transglycosylase Cwp19 and is most evident in glucose-containing BHI media and during later growth stages, while the second is a non-lytic mechanism controlled by TcdE, which is responsible for toxin release in earlier growth stages and in TY medium without glucose [21]. The more recent finding that the mutagenesis of *tcdE* in a different strain background reduced LCGT release provided further support for the role of TcdE in LCGT secretion [39]. However, the role of TcdE in toxin secretion continues to be debated because a mechanism by which a holin could release large proteins such as TcdA (308 kDa) and TcdB (270 kDa) is not apparent [27,41].

Here, we have shown that, similar to *C*. *difficile*, the PaLoc regions in other pathogenic clostridia such as *P. sordellii*, *C. perfringens*, and *C. novyii* encode a functional holin-like protein, although secretion via a holin-like protein was also shown for *C. perfringens* in a recent study [22]. We also show that the *P. sordellii* holin-like protein TcsE is clearly required for the efficient release of the toxin in strain ATCC 9714 (Figure 4a). While a lytic mechanism did also appear to partially release TcsL, the TcsE-mediated mechanism was unlikely to involve lysis, since no growth differences were detected between the relevant *tcsE* mutants when compared to the wild-type strain (Figure 4b). Therefore, while a specific TcsE-mediated pathway may be required for efficient TcsL release, it may be that an additional unrelated lytic mechanism is required to release enough TcsL. This additional lytic mechanism may be similar to that observed in *C. difficile* due to the protein Cwp19 [21], supported by the presence of proteins also with GHL10 (pfam02638) domains encoded on the chromosome (CEJ74920.1 in ATCC9714). These data suggest that the TcsE-mediated release of TcsL is a specific process, which was supported by results showing that there were no significant changes in the levels of the vast majority of cytoplasmic proteins identified in the secretomes of the *tcsE* mutant compared to the wild-type strain (Figure 5a, b). TcsL may not be the sole protein able to be secreted by TcsE, since the release of a λ phage endolysin, across the plasma membrane, was observed using an *E. coli* holin complementation system (Figure 3). However, in its natural host, TcsL appears to be the only protein that was significantly reduced in culture supernatants when TcsE is absent and is subsequently more abundant upon complementation of the *tcsE* mutation (Figure 5).

TcdE and, more recently, TpeE, which has been shown to mediate *C. perfringens* TpeL secretion independently of cell lysis [22], are perhaps the best studied of these secretion-related bacterial holin-like proteins. However, other studies have examined the role of bacterial holins in protein secretion. Among them, a holin-like protein, ChiW, appears to be required for the release of chitinases in *S. marcescens*. In fact, deletion of the encoding gene significantly reduced the release of three chitinases, a chitinase binding protein and at least six other proteins, although not all were confirmed by complementation [30]. Interestingly, it was noted that a cognate endolysin, ChiX, encoded within the same phage lysis-like locus, was also required for the release of these chitinases [30]. When ChiX was fused to a Tat signal peptide, thereby bypassing the need for holin-mediated translocation of the CM, the holin gene mutant was effectively complemented [30], indicating that the endolysin is the main facilitator of protein secretion, and that the holin is simply required to translocate the endolysin into the periplasmic space [30]. A non-phage associated endolysin-like protein, TtsA, also seems to control the release of typhoid toxin from *Salmonella* Typhi cells [38]. Recent work has shown that TstA is responsible for the transfer of Typhoid toxin from the *cis* to the *trans* side of the peptidoglycan, a process that, in turn, requires peptidoglycan remodelling by a cognate LD-transpeptidase [42]. In both of these examples, the endolysin-mediated release of proteins does not appear to be lytic. Instead, it was postulated that the endolysin functions to remodel the peptidoglycan within the cell wall to allow for the efficient transfer of specific proteins through the periplasm [30,38,42]. Whether an endolysin is required for the efficient release of the LCGTs from the clostridia is still a subject of debate.

It has been suggested that large proteins, such as LCGTs, are unlikely to be translocated via a holin alone. In fact, to do so, the holin would have to either actively transport these proteins or make a pore so large that cytoplasmic leakage or cell lysis would occur [27], although if toxins were transferred in an unfolded state, this leakage would be minimised. However, holins do not contain ATP-hydrolysis motifs, nor do the majority appear to function using proton-motive force [23,27]. The mechanism proposed by Saadat and Melville for TpeE-specific release of TpeL is a charged zipper mechanism, in which a cytoplasmic amphipathic helix on TpeE is activated by the insertion of a hydrophobic patch on TpeL, which then oligomerizes around TpeL to allow passage through the membrane. A high charge density domain at the C-terminus of TpeE (*KNKLNNN*) then acts to stabilise this confirmation [22]. While each of the other LCGTs also contain a hydrophobic patch that may initiate this system, an amphipathic helix is not predicted at the C-terminus of TcdE or TcsE [22]. This may mean that, while also requiring holin proteins for efficient secretion of LCGTs, the mechanism by which this is achieved differs between *C. perfringens* and the other clostridia.

The specific mechanism of secretion of these LCGTs is still up for debate and requires further investigation. It may be that the holin-like protein is responsible for the release of an intermediary protein that provides specificity for release. This intermediary protein may come in the form of an endolysin that remodels the peptidoglycan to allow the transit of such a large protein, as has been suggested in *S. marcescens* and *Salmonella* Typhi [30,38,42]. This hypothesis is consistent with the presence of endolysin-encoding genes in the PaLoc of *P. sordellii* and in some strains of *C. difficile* [37,43]. In this model, the specificity of release for TcsL that occurs in the presence of TcsE (Figure 5, Table 1) would be indirect, with TcsE required to specifically translocate an endolysin required to act and then facilitate the translocation of TcsL across the *P. sordellii* cell wall. However, this would still not address how the toxin is able to translocate across the cellular membrane. Alternatively, some version of the charged zipper mechanism suggested for TpeE/L may also be seen in the other Clostridia [22], where the insertion of the hydrophobic patch of the LCGT in the cell membrane is the trigger for oligomerisation around the toxin, preventing disruption of the proton motive force and resultant lysis from such a large pore. This explanation provides a pathway for a non-lytic, specific translocation of LGCTs across the cellular membrane. This model would also provide specificity of TcsE for TcsL, as suggested by our findings (Figure 5, Table 1), where it is the translocation across the cytoplasmic membrane that TcsE is required for and plays a direct role in. Regardless, the studies presented here suggest that TcsE-mediated secretion is specific for TcsL alone in *P. sordellii*, and that the *S*. *marcescens* ChiW and *C*. *difficile* TcdE systems also demonstrate some level of specificity [10,30], which means that cell lysis is probably not the release mechanism involved in these established holin-mediated systems [10].

Further analysis of LCGT secretion is therefore required. These studies will facilitate a deeper understanding of non-phage-associated holin-mediated secretion, which requires further investigation since they have been identified on the chromosomes of many Gram-negative and Gram-positive bacteria, as well as in archaea and eukaryotic organisms [24]. The specificity observed in the holin-mediated toxin release of TcsL from *P. sordellii*, detailed here, strongly supports the proposal of the existence of a new type of secretion system [44].

## 4. Materials and Methods

### 4.1. Bacterial Strains and Culture Conditions

Table S1 lists bacterial strains used in this analysis. Unless stated otherwise, clostridial species were cultured in HIS broth (37 g/L heart infusion broth (Oxoid), 5 g/L yeast extract, 1 g/L L-cysteine, 0.375% (wt/vol) glucose) or on HIS agar (HIS broth with 15 g/L agar) at 37 °C in an anaerobic chamber (Coy Laboratory Products, Inc., Grass Lake, MI, USA) in an atmosphere of 10% H2, 10% CO2, and 80% N2. *E. coli* strains were grown with shaking at 200 rpm in 2YT broth (16 g/L tryptone, 10 g/L yeast extract, 5 g/L NaCl) or on 2YT agar (2YT broth with 15 g/L agar) at 37 °C. When required, the following antibiotics were included for selection: erythromycin (10 mg/L), chloramphenicol (25 mg/L), thiamphenicol (10 mg/L), and lincomycin (50 mg/L).

### 4.2. Bioinformatic Analysis

Sequences representing the clostridial PaLocs and associated genome sequences were obtained from GenBank [45], with primary accession numbers for each sequence as follows: *C. difficile* 630 [46]: CP010905, *P. sordellii* ATCC 9714 [47]: LN679999, *P. sordellii* JGS6382 [47]: LN681235, *C. perfringens* JIR12708 [48]: LN835295 and *C. novyi* (A) BKT29909 [49]: JENM01000095. The sequences were further manually annotated using Artemis [50] on the basis of homology to entries in the Pfam database [35] via the HMMSCAN search on the HMMER web server [51]. The prediction of signal peptides and transmembrane domains within predicted amino acid sequences was performed using the Phobius web server [52] and confirmed using SignalP 4.1 [53]. Protein molecular weights were determined using SnapGene Viewer (from GSL Biotech; available at snapgene.com, accessed on 28 January 2022). The clostridial PaLocs were aligned, and the resulting graphics were produced using EasyFig [26]. Multiple sequence alignments were conducted using Clustal Omega from EMBL-EBI tools [54].

### 4.3. TcdE-like Holin Activity Assay

The *tpeE*, *tcsE*, and *tcnE* genes, including their own ribosomal binding site (RBS), were amplified by PCR (Table S2), digested and ligated to pBRQ(ΔRBS), a derivative of pJN4 deleted in the RBS of the λS gene [17]. To test the holin activity of the TcdE-like proteins, lysogens of *E. coli* strain MC1061 for a defective λ prophage bearing a nonsense mutation in its holin gene [λcI_857_Sam7] or carrying a deletion in holin and endolysin genes [λCm^r^Δ(*SR*)] were used as hosts for the plasmid constructs. These plasmids included pJN5, carrying the gene encoding the λ holin S105, and pBRQ(ΔRBS), used as positive and negative controls, respectively. Both λ(Sam7) and λCm^r^Δ(*SR*) encode a thermo-sensitive CI repressor (cI_857_) and are induced upon shifting the culture temperature from 30 °C to 42°C. Strains were grown in LB broth at 30 °C until the OD_600_ reached 0.15–0.25 before the thermo-induction of the λ prophage at 42 °C for 15 min. Bacterial growth and lysis at 37 °C were then followed by monitoring the absorbance at 600 nm at 15 min intervals. The curves in Figure 3 are a compilation of five independent cultures.

### 4.4. Mutagenesis

Mutagenesis using the TargeTron system and subsequent complementation was performed using a published method [55]. For mutagenesis, group II intron was retargeted to insert at the following distances from the predicted start of the coding sequence: between 161/162 bp of the anti-sense strand for ATCC 9714 *tcsE*, based on predictions from the Perutka Algorithm [56] via the ClosTron [57] design tool (http://clostron.com/clostron2.php, accessed on 10 March 2017). The correct insertion of the intron and the loss of the plasmid were confirmed using PCR and Southern hybridisation as previously described [55]. Primers used for PCR detection and probe generation in this study are listed in Table S2. TcsL TT mutant (DLL5002) referred to in the Supplementary Material was constructed as part of a previous study [58]. Similarly, the pCS1 plasmid-free *P. sordellii* strain referred to in the Supplementary Material was derived in a previous study, having lost the plasmid due to a targetron inactivation of the *parB* plasmid partitioning gene, followed by subculture [55].

### 4.5. Complementation in Trans

For complementation, genes including their RBS were PCR amplified using ATCC9714 genomic DNA as a template, with primers DLP525 and DLP526 for *tcsE*. PCR products were cloned into pRPF185 using restriction enzymes BamHI and SacI. The relevant primers are listed in Table S2.

### 4.6. TcsL Release Assays

Mid-logarithmic phase *P. sordellii* cultures in HIS broth were diluted to an OD_600_ of 0.3 before a 1/100 dilution into 20 mL TY (30 g/L tryptone, 20 g/L yeast extract) broth in tissue culture flasks. The TY cultures were incubated for 22 h at 37 °C in an anaerobic environment. At T = 5 h, anhydrous tetracycline (50 ng/mL) was added to all cultures to induce the complementation vectors. At T = 10 h, cells were collected and RNA extracted for transcript analysis. At T = 10 h and T = 22 h, culture supernatants were collected by pelleting cells by centrifugation at 2500× *g*, followed by filtering the supernatant through a 0.22-micron filter to remove all bacterial cells. The supernatants were analysed for TcsL levels using a *C. difficile* TcdB-specific ELISA detection kit (tgcBiomics), which is cross-reactive to TcsL (Figure S2), following the manufacturer’s instructions. TcsL levels were standardised to total protein based on a BCA assay (Thermo Scientific, Scoresby, Australia) and are presented relative to a TcdB standard curve. Statistical analysis was carried out using the Mann–Whitney *U* test on ≥4 independent biological replicates.

### 4.7. Vero Cell Cytotoxicity Assay

To prepare supernatants for cell cytotoxicity assays, *P. sordellii* was grown in 100 mL of TY broth with a starting optical density at 600 nm (OD600) nm of 0.05, for 24 h, and the cells pelleted by centrifugation. The supernatants were filter sterilized through 0.22 µ filters and stored at 4 °C prior to use. Cells were cultured in minimum essential medium (MEM alpha medium; GIBCO, Invitrogen, Scoresby, Australia) containing 10% heat-inactivated fetal calf serum (HI FCS), 100 units/mL penicillin, and 100 µg/mL streptomycin in culture flasks at 37 °C in 5% CO2. The cells were grown to a confluent monolayer and then sub-cultured by incubating in 2 mL of 0.1% trypsin in 1 mM EDTA. The cells were counted and resuspended in fresh medium at a concentration of 1 × 10^5^ cells/mL, and 100 µL aliquots of the cell suspension were seeded into each well of 96-well plates. The plates were then incubated for 20 h, followed by the removal of the culture medium. Serial two-fold dilutions of the *P. sordellii* culture supernatants were prepared in MEM alpha medium supplemented, with 1% HI FCS and 100 µL added to each well. Negative controls, which were cells treated with 100 µL MEM alpha medium supplemented with 1% HI FCS, were included. Plates were then incubated at 37 °C in 5% CO2 and morphological changes observed via microscopy after 24 h. The endpoint was scored as the last dilution at which a cytopathic effect (CPE) was observed.

### 4.8. Growth Curves

Mid-logarithmic phase *P. sordellii* cultures in HIS broth were diluted to an OD_600_ of 0.3 before a 1/100 dilution into 20 mL TY broth in glass bottles. The TY cultures were incubated for 22 h at 37 °C in an anaerobic environment. The OD_600_ of the cultures was recorded every two h from T = 1 to T = 11, with a final reading at T = 22.

### 4.9. Gene Expression Analysis

Total *P. sordellii* RNA was obtained from 3 mL of TY broth culture at T = 10 of the TcsL release assay, equating to an early stationary growth phase. RNA was isolated and its concentration determined using a published method [59]. The expression of *tcsL* in *P. sordellii* was determined by reverse-transcriptase digital droplet PCR (RT-ddPCR) based on a previous protocol [59]. All reactions were conducted as per the manufacturer’s (Bio-Rad) instructions. Briefly, 200 ng of total RNA was converted to cDNA using 100 ng of random primers (Promega) and SuperScript^®^ III reverse transcriptase (Invitrogen). PCR samples were set up to contain 5 ng or 0.5 ng of cDNA with genespecific primers at 200 nM each, along with an equal volume of QX200^TM^ddPCR^TM^EvaGreen Supermix (Bio-Rad, South Granville, Australia). PCR reactions were converted to droplets in DG8^TM^ cartridges (Bio-Rad) using the QX200^TM^ droplet generator (Bio-Rad) and amplified in a C1000 Touch^TM^ thermal cycler (Bio-Rad). Amplification events within individual droplets were measured by the QX200^TM^ droplet reader (Bio-Rad), and the resulting data were analysed using QuantaSoft^TM^ software (Bio-Rad). *tcsL* expression levels represent the number of transcripts per ng of cDNA, as a percentage of the transcripts of the housekeeping gene *rpoA*. Statistical analysis was carried out using the Mann–Whitney *U* test on four independent biological replicates.

### 4.10. Tryptic Digestion of Culture Supernatant Proteins

To concentrate *P. sordellii* secreted proteins, ice-cold 100% (*w*/*v*) tri-chloroacetic acid was added to 1 mL aliquots of filtered culture supernatant to achieve a final concentration of 10% (*v*/*v*). The supernatants were left on ice overnight, and precipitated proteins were then pelleted at 17,000× *g* for 30 min at 4 °C. The pellets were washed twice in 1 mL of −20 °C 90% (*v*/*v*) acetone by incubation on ice for 15 min followed by centrifugation for 30 min at 4°C. All remaining acetone was removed, and protein pellets were air-dried in a fume hood for 10 min and then stored at −80°C until needed.

Proteins were resuspended in 100 μL 1× LDS buffer (Thermo Fisher Scientific). The concentration of total protein (>10 kDa) in each sample was determined using densitometry. To do this, samples were separated by SDS-PAGE in a 4–15% gradient Stain-Free gel (Bio-Rad), and proteins in each lane were detected using the ChemiDoc Imaging system (Bio-Rad). Using Image Lab (Bio-Rad) software, densitometry against the BenchMark protein standard (Thermo Fisher Scientific) allowed protein concentrations to be determined. Samples were then diluted to 0.4 μg/ μL using 1× LDS buffer.

Then, 10 μg of protein from each sample (4 biological replicates) was simultaneously reduced and alkylated with 50 mM TCEP and 50 mM chloroacetamide, respectively, for 5 min at 95 °C. Proteins were then digested in-solution with 500 ng trypsin (Promega) and incubated overnight at 37 °C. Deoxycholate (DOC) was removed by phase transfer using an equal volume of ethyl acetate and acidified to 0.5% trifluoroacetic acid. The samples were then centrifuged at 14,000× *g* for 5 min, and the denser aqueous phase was collected and desalted using SDB-RPS StageTips [60]. The eluted peptides were evaporated to near dryness by vacuum centrifugation and suspended in ∼10 μL of 0.1% FA/2% ACN for proteomic analysis.

### 4.11. Mass Spectrometry Analysis and Protein Identification

The peptides (4 μL) were analysed by nanoliquid chromatography–tandem mass spectrometry on a Dionex Ultimate 3000 UPLC coupled to an Orbitrap Fusion Tribrid mass spectrometer (Thermo Fisher Scientific). Peptides were first loaded onto a trap column (Acclaim C18 PepMap nano Trap × 2 cm, 100 μm I.D, 5 μm particle size and 300 Å pore size; Thermo Fisher Scientific) at 15 μL min^−1^ for 3 min. The trap-column was then switched in line with the analytical column (Acclaim RSLC C18 PepMap Acclaim RSLC nanocolumn 75 μm × 50 cm, PepMap100 C18, 3 μm particle size, 100 Å pore size; ThermoFisher Scientific). Peptide separation was performed at 250 nl min^−1^ using reverse phase chromatography (Buffer A (0.1% FA, 2% ACN) and Buffer B (0.1% FA, 80% ACN). The non-linear gradient conditions for Buffer B were as follows: 2.5–7.5% over 1 min, 7.5–37.5% over 90 min, 37.5–42.5% over 3 min, 42.5–99% over 5 min, 99% hold 6 min, 99-2.5% over 1 min, and 2.5% hold 17 min. Data were collected in positive mode using data-dependent acquisition. MS scan properties were as follows: scan range (*m*/*z*) 375–1575, Orbitrap resolution 120,000, AGC target 1E6, maximum injection time 54 ms, and dynamic exclusion 15 s. MS/MS scan properties were as follows: HCD activation, isolation window of 1.8 (*m*/*z*), collision energy 32%, Orbitrap resolution 30,000, AGC target 2E5, maximum injection time 54 ms.

Raw files were analysed using the MaxQuant platform [61] version 1.5.5.1, searching against a concatenated database consisting of predicted *P. sordellii* ATCC9714 coding sequences from the available genome sequence (Genbank accession numbers LN679998, LN679999, and LN680000). Contaminant proteins from the media (yeast proteins (UniProt Proteome ID UP000002311) and casein subtypes (UniprotKB P02666, P02668, P02662 and P02663)) and products encoded by the Group II Intron or pRPF185 vector (ErmB (UniprotKB P20173) and β-glucuronidase (UniprotKB P05804)) were also identified but removed from further comparative analysis. Spectra were searched with the following criteria: specific digestion mode; maximum 2 missed cleavages; fixed modification of carbamidomethylcysteine; variable modifications of methionine oxidation; and N-terminal acetylation. Label-free quantification (LFQ), and match between run options were enabled, and false discovery rates of 1% for proteins and peptides were applied, with 2 minimum peptides required for identification. The raw data from this analysis are provided in Supplementary Data set 1.

### 4.12. Label-Free Quantitative Analysis of Culture Supernatant Proteins

Analysis of LFQ data was performed using Perseus version 1.5.6.0 [62]. Identified proteins were removed from the dataset if they were not detected in at least 3 out of 4 replicates from at least one strain. The LFQ values were normalised using a log_2_ transformation. Non-valid values (below the limit of detection) were inputted through random selection of values within ±0.2 standard deviations from the mean of the Gaussian distribution of the data set for their respective biological replicate, having first being shifted 1.9 standard deviations to the left. Statistical analysis of LFQ values for two strains for a particular protein was performed using a 2-tailed student’s t-test. For screening purposes, the statistical significance cut-off was considered a permutation-based false discovery ratio of 0.1 using 250 randomisations, while implementing an artificial within group’s variance of 0.1.

## Figures and Tables

**Table 1:** Proteins significantly altered in quantity between the culture supernatants of (**a**) *tcsE* mutant (M) and wildtype (WT) *P. sordellii* ATCC9714 strains and (**b**) complemented *tcsE* mutant (C) and the *tcsE* mutant vector control (VC) *P. sordellii* ATCC9714 strains.

(a)		M vs. WT			C vs. VC		
Protein	Protein ID	Fold Change	*p*-Value (×10^−3^)	q-Value	Fold Change	*p*-Value (×10^−^^3^)	q-Value
50S ribosomal protein L31	CEJ72251.1	0.12	0.180	0.224	0.70	541	0.679
50S ribosomal protein L24	CEJ72165.1	0.38	0.847	0.126	1.06	648	0.836
**TcsL—Lethal Toxin**	**CEJ75463.1**	**0.48**	0.641	0.111	**2.25**	1.73	0.147
50S ribosomal protein L5	CEJ72166.1	0.51	1.17	0.103	0.96	779	0.896
probable polysaccharidede acetylase	CEJ75226.1	0.51	1.41	0.104	0.90	626	0.802
50S ribosomal protein L10	CEJ72145.1	0.53	1.27	0.092	0.80	314	0.547
50S ribosomal protein L22	CEJ72159.1	0.54	0.122	0.131	0.79	200	0.435
50S ribosomal protein L4	CEJ72155.1	0.62	0.340	0.106	0.66	152	0.353
phospholipase D-nuclease domain protein	CEJ72911.1	4.69	0.967	0.170	2.47	26.2	0.158
**(b)**		**C vs. VC**			**M vs. WT**		
**Protein**	**Protein ID**	**Fold Change**	***p*** **-Value** **(×10** **^−3^** **)**	**q-Value**	**Fold Change**	***p*** **-Value** **(×10** **^−3^** **)**	**q-Value**
spore coat peptide assembly protein CotJC2	CEJ72697.1	2.07	1.586	0.136	0.91	753	0.913
**TcsL—Lethal Toxin**	**CEJ75463.1**	**2.25**	1.731	0.147	**0.48**	0.641	0.111
branched-chain amino acid aminotransferase	CEJ74628.1	2.29	3.446	0.092	0.94	560	0.896
putative delta-lactam-biosynthetic de-N-acteylase	CEJ73318.1	2.37	3.296	0.123	2.44	5.69	0.138
putative ferredoxin/flavodoxinoxido reductase, alpha subunit	CEJ75209.1	2.48	1.890	0.169	0.95	702	0.914
putative pyridine nucleotide-disulphide oxidoreductase	CEJ73686.1	2.62	4.003	0.111	1.09	653	0.899
dimethylamine corrinoid protein	CEJ74389.1	2.77	0.808	0.131	2.50	132	0.643
putative ferredoxin/flavodoxinoxido reductase, beta subunit	CEJ75208.1	2.86	4.135	0.139	1.15	402	0.896
hypothetical protein	CEJ73866.1	3.77	0.002	0.068	1.23	639	0.881
Chorismite mutase	CEJ73786.1	4.10	7.106	0.085	0.82	776	0.914
conserved hypothetical protein	CEJ72332.1	5.12	0.711	0.138	3.47	14.7	0.185
hypothetical protein	CEJ74906.1	6.91	8.456	0.101	1.40	766	0.911
ureE urease accessory, N-terminal domain protein	CEJ73734.1	6.93	1.349	0.132	0.88	571	0.892

The proteins and their protein ID accession numbers are listed in the order of fold change. Given are the average fold change, *p*-value, and q-value determined by a two-tailed student t-test of log_2_ transformed intensity values, obtained by mass spectrometry-based label-free protein quantification. The value for the comparison of the (a) complemented *tcsE* mutant (C) to the *tcsE* mutant containing the vector control (VC), or (b) *tcsE* mutant (M) to the wildtype (WT), for these proteins is also provided. The only protein identified to be altered in both comparisons, and its corresponding values, is shown in bold.

## Data Availability

Not applicable.

## Supplementary Materials

The following are available online at https://www.mdpi.com/article/10.3390/toxins14020124/s1, Figure S1: Confirmation of the insertional inactivation of *tcsE* of *P. sordellii* ATCC9714. Confirmation of independent *tcsE* mutants of *P. sordellii* ATCC9714 were carried out using Southern hybridization with PstI digested genomic DNA. The blots were hybridized with DIG-labelled DNA probes specific for (a) *tcsE*, (b) *ermB*, carried within the group II intron, and (c) *catP*, carried on the TargeTron vector backbone. V: HindIII digested re-targeted pDLL46 vector; M: DIG-labelled λ-HindIII molecular size markers (labelled in kb); WT: wild-type isolate. The TargeTron insertion site is present within a 3912 bp fragment on the WT genome producing an ~5712 bp fragment in correct mutants., Figure S2: A TcdB ELISA that specifically measures TcsL in culture supernatant. Mid-log HIS broth cultures of an isogenic panel of *P. sordellii* strains were diluted to an OD_600_ of 0.3 before a 1/100 dilution into TY broth and incubated at 37 °C in an anaerobic environment for 22 h. The culture supernatants were collected and assayed using a TcdB-specific ELISA detection kit (tgcBiomics). Included in the analysis were the wild-type isolate ATCC 9714 (WT), an insertional inactivation mutant of the *tcsL* gene (*tcsL*TT) and a pCS1-1 (the plasmid which carried *tcsL*)-free version of ATCC 9714 (pCS1-1^−^) obtained as part of a previous study [55,58]. Shown are the absorbance values (450 nm–620 nm) that correlate with TcdB levels, as per the manufacturer’s protocol. Absorbance values for decreasing concentrations of purified TcdB (tgcBiomics) are included, along with the average values for 3 biological replicates of *P. sordellii* supernatant. Error bars represent SEM. Figure S3: TcsL toxin levels of supernatants from isogenic strains harvested at 10 h. At T = 10 h culture supernatants were collected and assayed using a TcdB-specific ELISA detection kit (tgcBiomics). The concentrations of TcsL detected relative to a TcdB standard protein curve are shown, having been standardised to the total protein concentration in the supernatant, as determined by BCA assays. n ≥ 2, error bars indicate SEM. Figure S4: Vero cell cytotoxicity assay. Serial doubling dilutions of *P. sordellii* culture supernatants were produced in MEM alpha medium supplemented with 1% HI FCS and used in cytotoxicity assays. Changes in Vero cell morphology were then scored by microscopy after 24 h. The endpoint was taken as the last dilution at which CPE was observed. The toxin titer represents the reciprocal of the endpoint dilution. WT, wild-type ATCC 9714; *tcsE1*, *tcsE* mutant strain DLL5036; *tcsE1*(V) is DLL5240, the *tcsE* mutant strain carrying the vector control, *tcsE1*(*tcsE*+) is DLL5159, the complemented *tcsE* mutant. n = 1. Table S1: Bacterial isolates and plasmids used in this study. Table S2: Oligonucleotide primers used in PCR and Supplementary File S1: Mass spectrometry data set used for Table 1 and Figure 5.
