# Supplementary material for: A Highly Specific Holin-Mediated Mechanism Facilitates the Secretion of Lethal Toxin TcsL in Paeniclostridium sordellii
Source: Toxins (Basel). 2022 Feb 8;14(2):124. doi: 10.3390/toxins14020124 (PMC8878733; doi:10.3390/toxins14020124)
Supplement: Supplementary file 1 [file toxins-14-00124-s001.zip › toxins-1543634-supplementary.pdf]

Supplementary Materials: A Highly Specific Holin-Mediated Mechanism Facilitates the Secretion of Lethal Toxin TcsL in *Paeniclostridium sordellii*

Callum J. Vidor, Audrey Hamiot, Jessica Wisniewski, Rommel Mathias, Bruno Dupuy, Milena Awad and Dena Lyras

Table S1. Bacterial isolates and plasmids used in this study.

Strain/ plasmid	Characteristics	Reference
	P. sordellii	
ATCC9714	Bovine, myonecrosis, contains pCS1-3, *tcsH*$^-$*tcsL*$^+$	[1]
DLL5002 [*tcsL*TT]	ATCC9714Ω*tcsL*::TT, Em^R	[2]
DLL5143 [pCS1-1$^-$]	ATCC9714 cured of pCS1-1, *tcsL*$^-$	This study, derived from [3].
DLL5036 [*tcsE*TT1]	ATCC9714Ω*tcsE*::TT, independent mutant 1, Em^R	This study
DLL5037 [*tcsE*TT2]	ATCC9714Ω*tcsE*::TT, independent mutant 2, Em^R	This study
DLL5159 [*tcsE*TT1(*tcsE*$^+$)]	DLL5036(pDLL101), Em^R, Tm^R	This study
DLL5240 [*tcsE*TT1(V)]	DLL5036(pRPF185), Em^R, Tm^R	This study
	E. coli	
MC1061/ λcI$_{857}$Sam7	Lysogenic MC1061 carrying λcI$_{857}$Sam7, does not encode a functional holin	[4]
MC1061/ λCm^{r}Δ(*SR*)	Lysogenic MC1061 carrying λCm^{r}Δ(*SR*), does not encode a functional holin or endolysin	[5]
DH5α	F$^-$ φ80*lac*ZΔM15 Δ(*lac*ZYA-*arg*F)U169 *rec*A1 *end*A1 *hsd*R17(r$_{K^-}$, m$_{K^+}$) *pho*A *sup*E44 λ$^-$ *thi*-1 *gyr*A96 *rel*A1, used for subcloning	Thermo Fisher Scientific
HB101(pVS520)	*recA123*, Res$^-$, Mod$^-$, Str^R containing pVS520, Tc^R, used as conjugation donor for *P. sordellii*	[6]
	**Plasmids**	
pBRQ(ΔRBS)	pJN4 derivate; λS gene with a deletion in its ribosome binding site (RBS) under the control of the late transcription regulatory (LTR) elements of phage λ	[7]
pJN5	pBR322 derivative; carries S105 under the control of the LTR elements of phage λ	[8]
pRG32	pBRQ(Δrbs) carrying *tcdE* with its own RBS	[7]
pDIA6884	pBRQ(Δrbs) carrying *tcsE* with its own RBS	This study
pDIA6883	pBRQ(Δrbs) carrying *tpeE* with its own RBS	This study
pDIA6885	pBRQ(Δrbs) carrying *tcnE* with its own RBS	This study
pDLL46	Clostridial TargeTron vector, contains RP4 and Tn*916 oriT*s and *lacZα* within retargeting region for blue white screening	[3]
pVS520	Tra$^+$, Mob$^+$, RP1 derivative	[6]
pRPF185	Clostridial tetracycline inducible expression vector	[9]
pDLL101	pRPF185 carrying *tcsE* with its predicted RBS under a tetracycline inducible promoter	This study

Table S2. Oligonucleotide primers used in PCR.

Primer	Sequence (5′-3′)	Use
OBD778	GCTCTAGATGAATATAACAATATCTTTTTTATCAAAA	Amplify *tcsE* and predicted RBS (+) for cloning into pBRQ(ΔRBS)
OBD779	CCCAAGCTTCATTATTTTTATCTATCCTCAATTTTTAC	Amplify *tcsE* and predicted RBS (-) for cloning into pBRQ(ΔRBS)
OBD776	GCTCTAGAGTGGATTCAGAACTGTTTAAAATTATG	Amplify *tpeE* and predicted RBS (+) for cloning into pBRQ(ΔRBS)
OBD777	CCCAAGCTTCTAATTATTATTCAATTTATTTTTTATGTCTTC	Amplify *tpeE* and predicted RBS (-) for cloning into pBRQ(ΔRBS)
OBD780	GCTCTAGAATGGATAAACAGAATATTTTTAACAC	Amplify *tcnE* and predicted RBS (+) for cloning into pBRQ(ΔRBS)
OBD781	CCCAAGCTTGCAAGACTATTTGTCCTGCTC	Amplify *tcnE* and predicted RBS (-) for cloning into pBRQ(ΔRBS)
JRP3867	CGAAATTAGAAACTTGCGTTCAGTAAAC	TargeTron EBS universal
DLP965	AAAAAAGCTTATAATTATCCTTAAATATCCAAGCTGTGCGCCCAGATAGGGTG	*tcsE* TargeTron IBS, 161/162 antisense
DLP966	CAGATTGTACAAATGTGGTGATAACAGATAAGTCCAAGCTATTAACTTAC-CTTTCTTTGT	*tcsE* TargeTron EBS1d, 161/162 antisense
DLP967	TGAACGCAAGTTTCTAATTTCGGTTATATTCCGATAGAGGAAAGTGTCT	*tcsE* TargeTron EBS2, 161/162 antisense
DLP525	AAAGAGCTCCTAGGAGGCATTATGAATATAAC	Amplify *tcsE* and predicted RBS (+) for complementa-tion - introduces SacI site, screening of mutants, gener-ation of probe
DLP526	AAAGGATCCATATTTTCATTATTTTTATCTATCCTC	Amplify *tcsE* and predicted RBS (-) for complementation - introduces BamHI site, screening of mutants, gener-ation of probe
DLP968	AAGAACTCAGCGAAACAAATGAC	Internal *tcsL* (+), RT-ddPCR
DLP969	TTACTAAACTTGGTATCCCTGCTG	Internal *tcsL* (-), RT-ddPCR
DLP970	GAAGCACAAGGACCATGTACAG	Internal *P. sordellii rpoA* (+), RT-ddPCR
DLP971	CTTCCTTTATTCACAGATATTTCCATG	Internal *P. sordellii rpoA* (-), RT-ddPCR

(+) forward primer, (-) reverse primer.

References

1. Hall, I.C., Scott, J.P. *Bacillus sordellii*, a Cause of Malignant Edema in Man. *J. Infect. Dis.* **1927**, *41*, 329–335.
2. Carter, G.P.; Awad, M.M.; Hao, Y.; Thelen, T.; Bergin, I.L.; Howarth, P.M.; Seemann, T.; Rood, J.I.; Aronoff, D.M.; Lyras, D. TcsL is an Essential Virulence Factor in *Clostridium sordellii* ATCC 9714. *Infect. Immun.* **2011**, *79*, 1025–1032, doi:10.1128/iai.00968-10.
3. Vidor, C.J.; Watts, T.D.; Adams, V.; Bulach, D.; Couchman, E.; Rood, J.I.; Fairweather, N.F.; Awad, M.; Lyras, D. *Clostridium sordellii* Pathogenicity Locus Plasmid pCS1-1 Encodes a Novel Clostridial Conjugation Locus. *mBio* **2018**, *9*, doi:10.1128/mBio.01761-17.
4. Goldberg, A.R.; Howe, M. New Mutations in the S Cistron of Bacteriophage Lambda Affecting Host Cell Lysis. *Virology* **1969**, *38*, 200–202.
5. Smith, D.L.; Young, R. Oligohistidine Tag Mutagenesis of the lambda Holin Gene. *J. Bacteriol.* **1998**, *180*, 4199–4211.

6. Palombo, E.A.; Yusoff, K.; Stanisich, V.A.; Krishnapillai, V.; Willetts, N.S. Cloning and Genetic Analysis of *tra* Cistrons of the Tra 2/Tra 3 Region of Plasmid RP1. *Plasmid* **1989**, *22*, 59–69, doi:http://dx.doi.org/10.1016/0147-619X(89)90036-X.
7. Govind, R.; Dupuy, B. Secretion of *Clostridium difficile* Toxins A and B Requires the Holin-Like Protein TcdE. *PLoS Pathog.* **2012**, *8*, e1002727.
8. Sao-Jose, C.; Santos, S.; Nascimento, J.; Brito-Madurro, A.G.; Parreira, R.; Santos, M.A. Diversity in the Lysis-Integration Region of Oenophage Genomes and Evidence for Multiple tRNA Loci, as Targets for Prophage Integration in *Oenococcus oeni*. *Virology* **2004**, *325*, 82–95, doi:10.1016/j.virol.2004.04.029.
9. Fagan, R.P.; Fairweather, N.F. *Clostridium difficile* has Two Parallel and Essential Sec Secretion Systems. *J. Biol. Chem.* **2011**, *286*, 27483–27493, doi:10.1074/jbc.M111.263889.
