# Supplementary figures and images for: A Highly Specific Holin-Mediated Mechanism Facilitates the Secretion of Lethal Toxin TcsL in Paeniclostridium sordellii
Source: Toxins (Basel). 2022 Feb 8;14(2):124. doi: 10.3390/toxins14020124 (PMC8878733; doi:10.3390/toxins14020124)

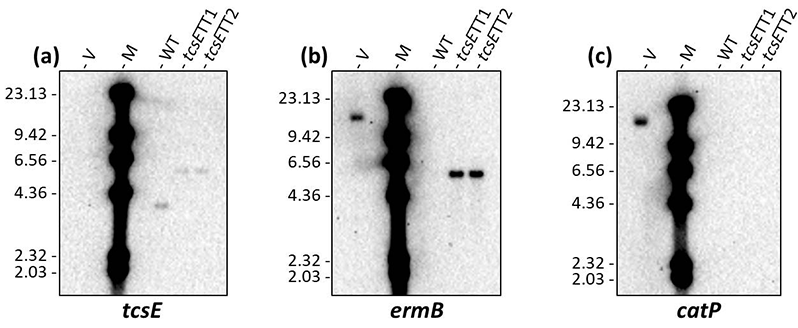

Supplement: Supplementary file 1 [file toxins-14-00124-s001.zip › Figure S1.tif]

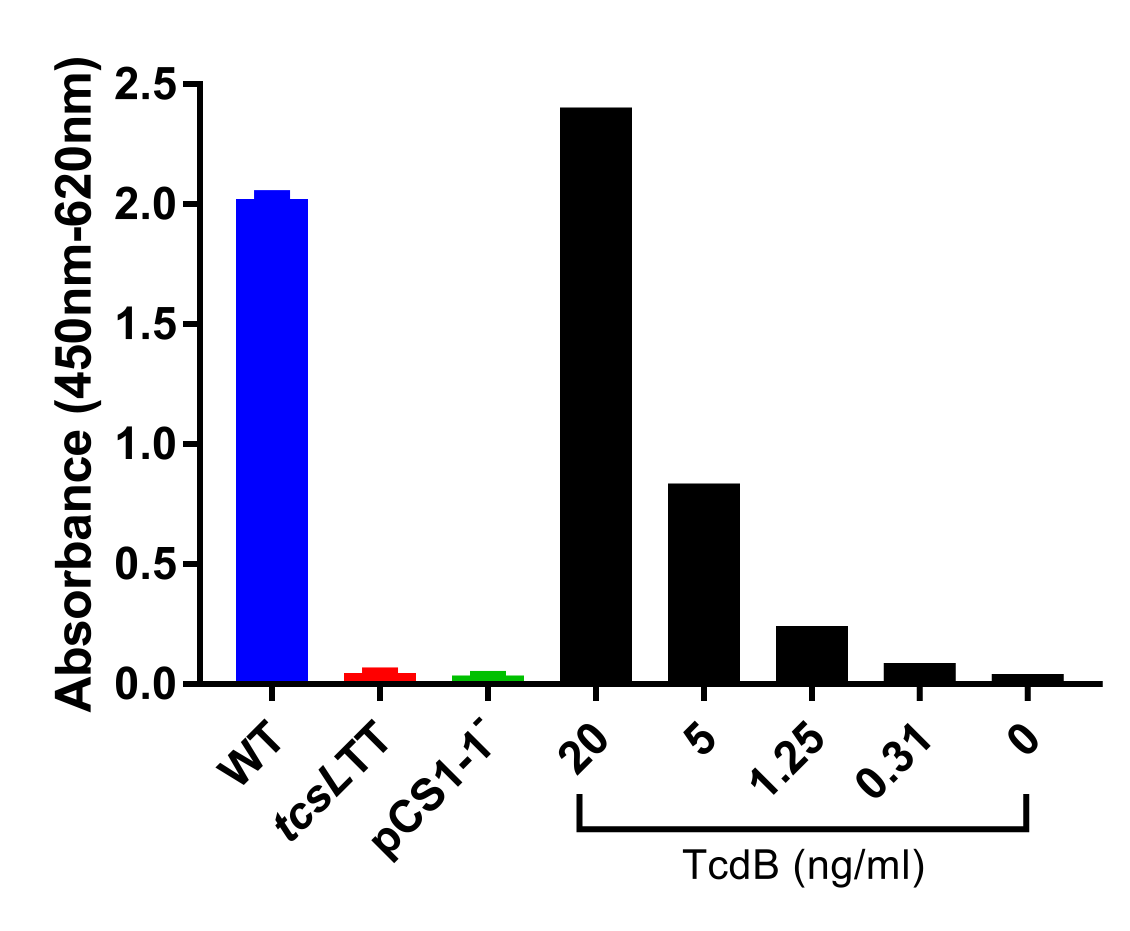

Supplement: Supplementary file 1 [file toxins-14-00124-s001.zip › Figure S2.tif]

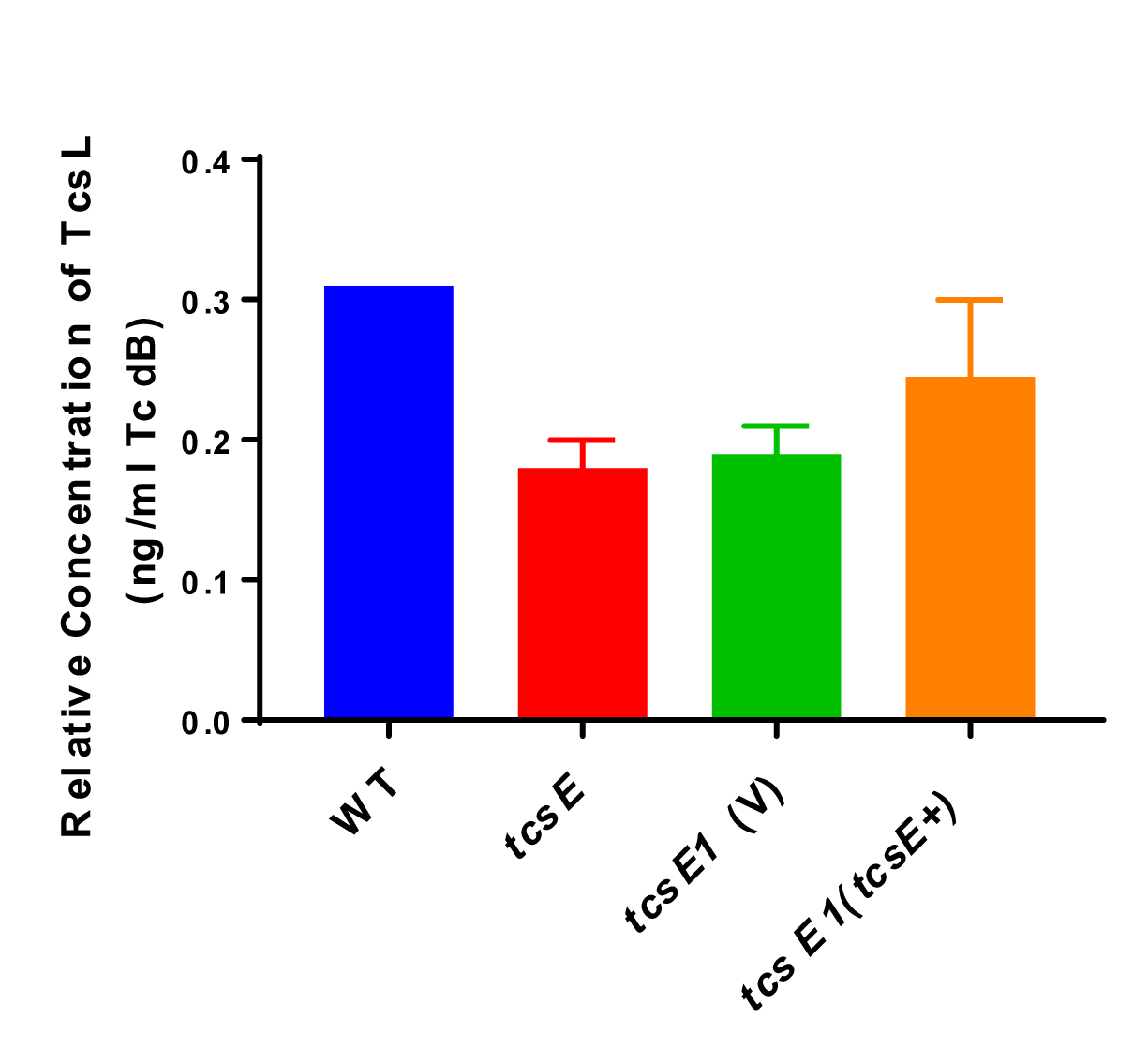

Supplement: Supplementary file 1 [file toxins-14-00124-s001.zip › Figure S3.tif]

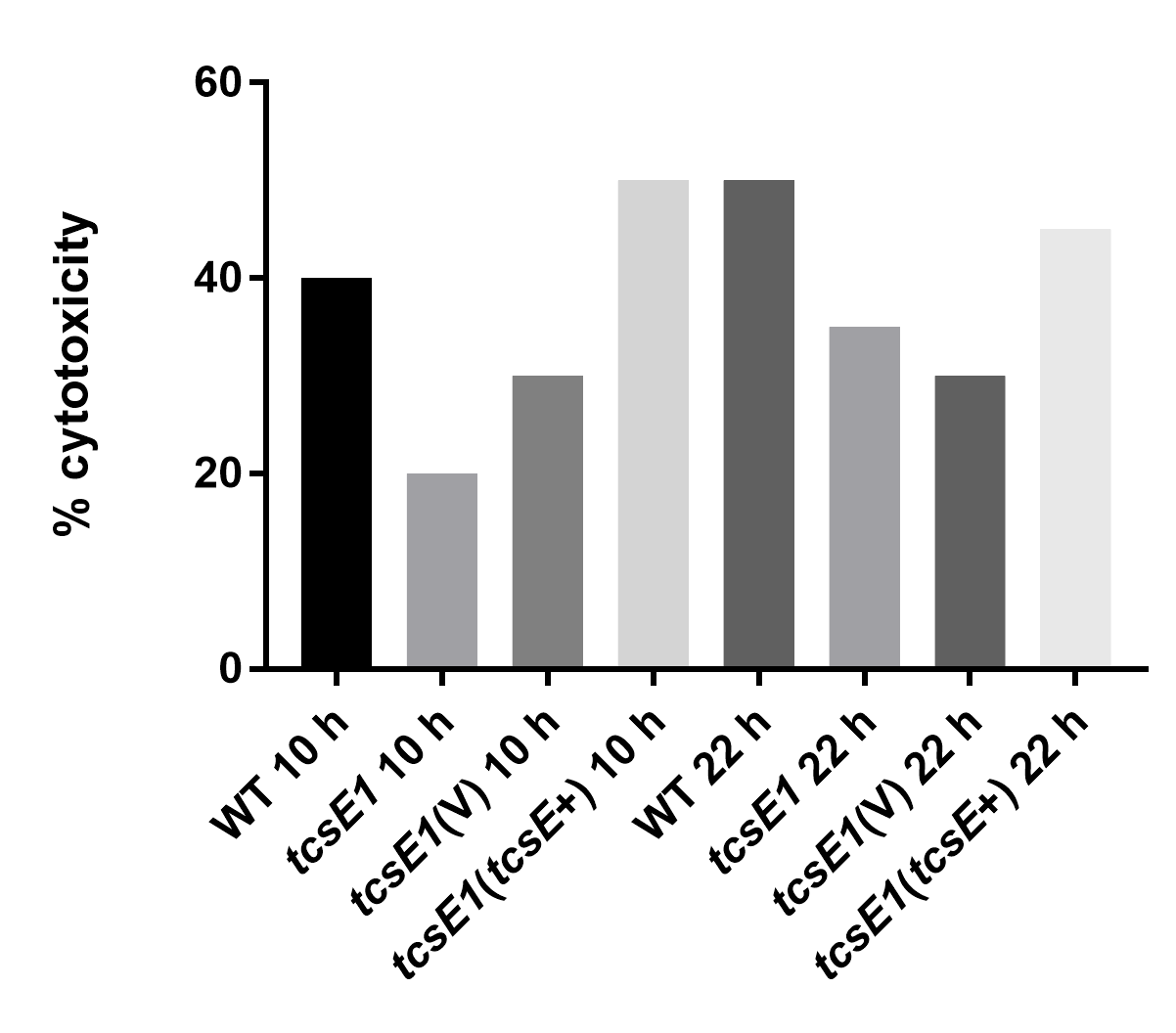

Supplement: Supplementary file 1 [file toxins-14-00124-s001.zip › Figure S4.tif]
